# Socio-economic deprivation and COVID-19 in Germany

**DOI:** 10.1177/14034948221080397

**Published:** 2022-03-23

**Authors:** Angela P. Moissl, Stefan Lorkowski, Winfried März

**Affiliations:** 1 Institute of Nutritional Sciences, Friedrich Schiller University Jena, Germany; 2 Competence Cluster for Nutrition and Cardiovascular Health (nutriCARD) Halle-Jena-Leipzig, Germany; 3 Medical Faculty Mannheim, University of Heidelberg, Germany; 4 Clinical Institute of Medical and Chemical Laboratory Diagnostics, Medical University Graz, Austria; 5 Synlab Academy, Synlab Holding Deutschland GmbH, Germany

## Introduction

In 2020, SARS-CoV-2 spread worldwide after it initially appeared in Wuhan, China, in December 2019. To try to prevent the spread to countries with poor infrastructure and poor health-care systems, the World Health Organization (WHO) classified SARS-CoV-2 as a pandemic and recommended social distancing and mask wearing to slow infection rates. According to some studies, countries with elderly populations or underdeveloped health-care systems have been more affected than others [1]. The role of the socio-economic environment as an important factor has been also addressed [2].

Studies from the USA and China show that the acceptance of protective measures and infection rates correlated with socio-economic status and education levels [3,4]. Considering the possible socio-economic gradient, we hypothesised that the number of infections could be directly linked to the German Index of Socioeconomic Deprivation (GISD).

## Methods

The GISD was developed by Kroll et al. and describes regional socio-economic deprivation in Germany [5]. It summarises the extent of the socio-economic disadvantages of regions by considering education, employment and income. The higher the GISD, the higher the deprivation [5]. Case numbers, deaths and seven-day incidence rates were obtained from the Robert Koch Institute (RKI). The RKI receives its data from the health authorities of the individual districts, which transmit their current data as daily reports [6].

Based on international standards of the WHO, the SARS-CoV-2 cases at the RKI were independent of the presence or severity of clinical symptoms rated as COVID-19 cases. The seven-day incidence rates describe the sum of the number of confirmed infections in the last seven days divided by the number of residents per district (based on most recent data provided publicly by the German Federal Statistical Office (destatis.de) [7] on 31 December 2019) and is normalised to 100,000 people [6].

We retrieved the seven-day incidence rates of SARS-CoV-2 infections per 100,000 inhabitants from 18 November 2020 to 18 January 2021 at the district level and correlated it with the GISD. We then calculated the median cumulative seven-day incidence rates for three selected representative days (18 November 2020, 18 December 2020 and 18 January 2021). All calculations, analyses and maps were carried out with R v4.0.4 (The R Foundation for Statistical Computing, Vienna, Austria).

## Results

**Figure 1.**
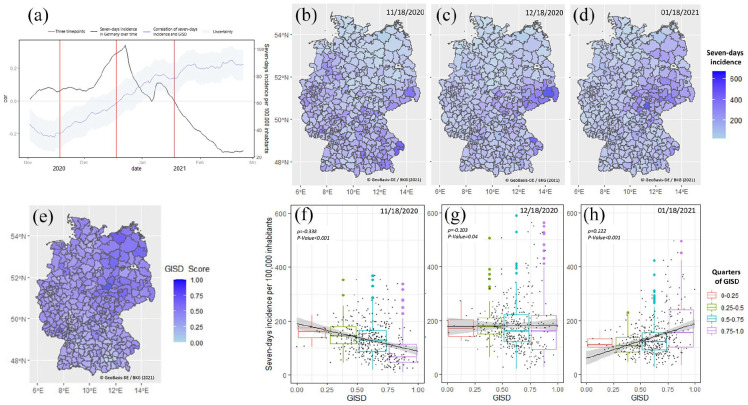
Changes in the correlation of the German Index of Socioeconomic Deprivation (GISD) with seven-day incidence rates of SARS-CoV-2 infections per 100,000 inhabitants. (a) Seven-day incidence rates of SARS-CoV-2 infections per 100,000 inhabitants and correlations of seven-day incidence rates and GISD over time in Germany are shown as a black line. The correlation of the GISD and the seven-day incidence rates with confidence intervals is shown in blue. The three selected days are marked as red lines. (b)–(d) Seven-day incidence rates of SARS-CoV-2 infections per 100,000 inhabitants in Germany at the district level at selected dates as indicated. (e) Distribution of the GISD at district level in Germany: the darker the colour, the higher the GISD. (f)–(h) Correlation of seven-day incidence rates of SARS-CoV-2 infections per 100,000 inhabitants with the GISD at district level shown in quarters at selected dates as indicated.

Distribution of the GISD is shown in Figure 1(e). In Figure 1(b)–(d), seven-day incidence rates of SARS-CoV-2 infections per 100,000 inhabitants at the district level are shown across Germany in the said period for three selected representative days. The corresponding correlations are shown in Figure 1(f)–(h). Interestingly, correlations between incidence rates and GISD changed over time. While on 18 November 2020 incidence rates were higher in areas with low GISD, this correlation disappeared within the following four weeks and showed a uniform distribution of incidence rates on 18 December 2020. Another four weeks later, the correlation turned to the opposite, with higher incidence rates in areas with higher GISD.

At the beginning of our observation period (18 November 2020), seven-day incidence rates were in the lowest quarter of the GISD (0–0.25) with a median of 162 (95% confidence interval (CI) 138–175), while seven-day incidence rates in the highest quarter (0.75–1.0) were at a median of 67 (95% CI 10–48). Between 18 November 2020 and 18 December 2020, the seven-day incidence rates shifted. In the first quarter of the GISD, seven-day incidence rates remained high with a mean median of 171 (95% CI 141–204) and increased to a median of 167 (95% CI 95–222) in the fourth quarter. On 18 January 2021, seven-day incidence rates in the first GISD quarter declined to a median of 111 (95% CI 103–132) and remained at a median of 156 (95% CI 102–241) in the fourth quarter. Over the same period, a shift in seven-day incidence rates from south-western to north-eastern Germany could be observed (Figure 1(b)–(d)).

The high seven-day incidence rates on 18 December 2020 in all quarters of the GISD reflect the changes in the overall seven-day incidence rates in Germany in this period, which were as follows: 138.9 (18 November 2020), 184.8 18 (December 2020) and 134.4 (18 January 2021). Interestingly, the median of the seven-day incidence rates of the highest GISD quarter never exceeded that of the lowest GISD quarter in the studied period. To compare the data over time, Figure 1(a) shows the seven-day incidence rates between 18 November 2020 and 18 February 2021 as a median curve for Germany as a black line. The correlation of the GISD and the seven-day incidence rates is shown in blue in Figure 1(a). The three selected days are marked as red lines. Supplemental Figures 1 and 2 show the time series from 18 November 2020 to 20 January 2021 that clearly demonstrates the continuous change in the correlation of the GISD and the seven-day incidence rates at the district level.

## Discussion

On 23 March 2021, the RKI reported socio-economic differences in the risk of infection during the second SARS-CoV-2 wave in Germany [8]. The authors concluded that areas with higher GISD are more affected by SARS-CoV-2 infections than areas with lower GISD. Alternatively, we hypothesise that the pendulum-like changes in the correlation of the GISD and seven-day incidence rates may depend more on the spatiotemporal spread of SARS-CoV-2 from south-western to north-eastern Germany over the reported period than on social status.

Furthermore, there is no clear correlation between the nationwide cumulative seven-day incidence rates and the GISD. In contrast, we even observe contradictory correlations, depending on the different phases of the spread of the virus in Germany. Likely due to the uneven spatial distribution of SARS-CoV-2 infections, the temporal and geographical course of the pandemic, test strategies and other external influencing factors such as people’s mobility, no direct association of incidence rates with the GISD can be inferred.

We conclude that associations of incidence rates of infections with socio-economic or sociodemographic indices should always be interpreted carefully, as the spatiotemporal spread of virus infections may preface misleading associations.

### Limitations

Data on SARS-CoV-2 infections and socio-economic status at the individual level were not available. Therefore, only aggregated data have been used for the analyses.

## Supplemental Material

sj-png-1-sjp-10.1177_14034948221080397 – Supplemental material for Socio-economic deprivation and COVID-19 in Germany Click here for additional data file. Supplemental material, sj-png-1-sjp-10.1177_14034948221080397 for Socio-economic deprivation and COVID-19 in Germany by Angela P. Moissl, Stefan Lorkowski and Winfried März in Scandinavian Journal of Public Health

sj-png-2-sjp-10.1177_14034948221080397 – Supplemental material for Socio-economic deprivation and COVID-19 in Germany Click here for additional data file. Supplemental material, sj-png-2-sjp-10.1177_14034948221080397 for Socio-economic deprivation and COVID-19 in Germany by Angela P. Moissl, Stefan Lorkowski and Winfried März in Scandinavian Journal of Public Health
