# Supplementary figures and images for: Socio-economic deprivation and COVID-19 in Germany
Source: Scand J Public Health. 2022 Mar 23;50(6):668–70. doi: 10.1177/14034948221080397 (PMC9361408; doi:10.1177/14034948221080397)

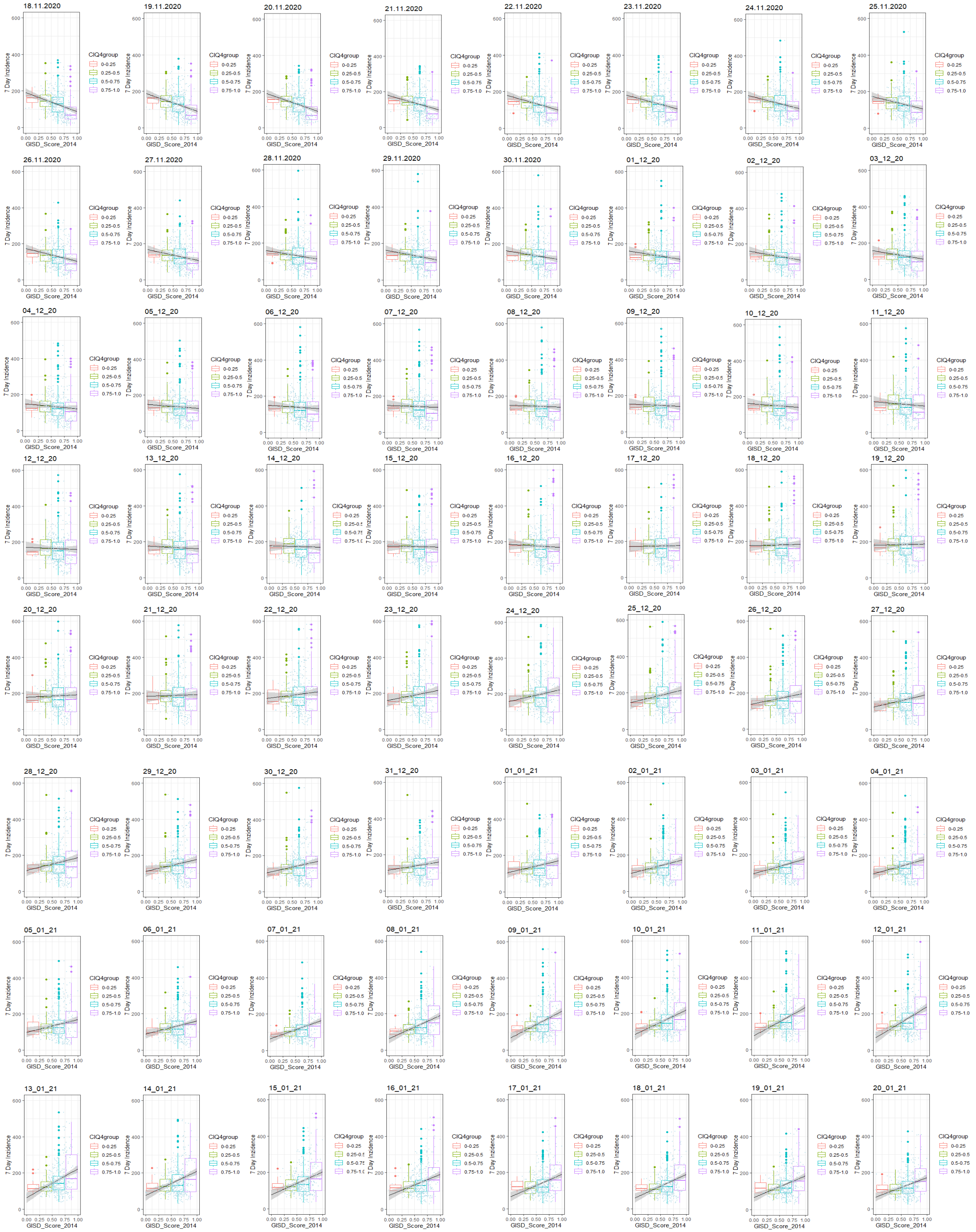

Supplement: sj-png-1-sjp-10.1177_14034948221080397 – Supplemental material for Socio-economic deprivation and COVID-19 in Germany [file sj-png-1-sjp-10.1177_14034948221080397.png]

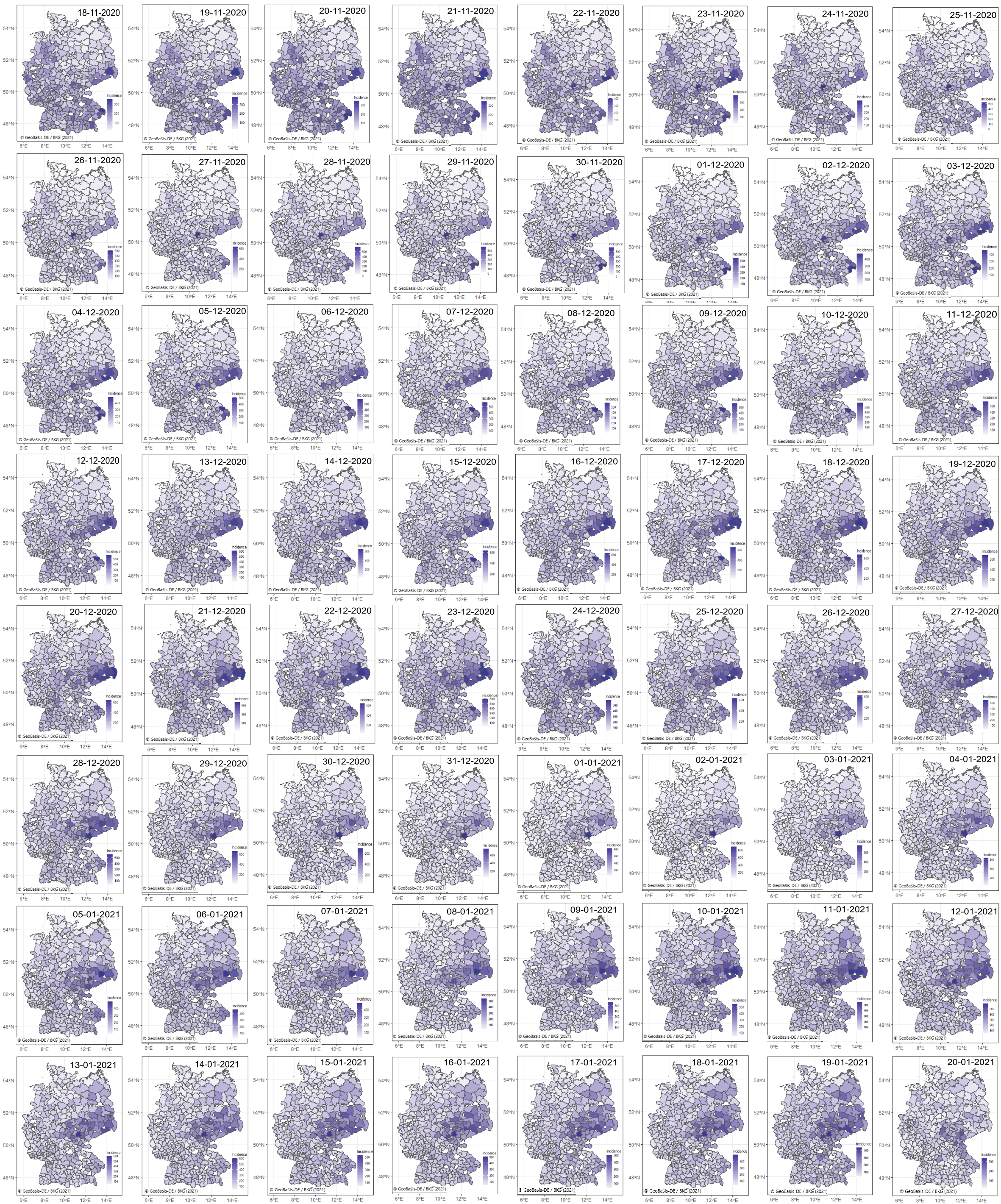

Supplement: sj-png-2-sjp-10.1177_14034948221080397 – Supplemental material for Socio-economic deprivation and COVID-19 in Germany [file sj-png-2-sjp-10.1177_14034948221080397.png]
